# A Case of Pediatric Breast Abscess Caused by Rarely Observed Bacteria in a Three-Year-Old Boy With an Inverted Nipple: Peptoniphilus harei, Actinotignum sanguinis, and Porphyromonas somerae

**DOI:** 10.7759/cureus.41011

**Published:** 2023-06-26

**Authors:** Manami Ueshima, Satoshi Matsuda, Mayumi Iwama

**Affiliations:** 1 Department of Pediatrics, Kameda Medical Center, Kamogawa, JPN; 2 Department of Pediatric Surgery, Kameda Medical Center, Kamogawa, JPN

**Keywords:** child, infection, inverted nipple, mastitis, breast abscess

## Abstract

Mastitis and breast abscesses are most common in lactating women but can also be observed in non-lactating women, adolescent girls, and neonates. However, breast abscesses are extremely rare in young boys. Herein, we report the case of a three-year-old boy with a swollen and painful right nipple, later diagnosed with a breast abscess. In this case, we suspected that the patient’s inverted nipple was the possible site of the infection. To our best knowledge, this is the first case report of breast abscess in a young boy after the neonatal period. Although *Staphylococcus aureus* is the most common pathogen, our patient showed three rare bacteria, namely, *Peptoniphilus harei*, *Actinotignum sanguinis*, and *Porphyromonas somerae*, in the culture of the aspirated pus. Furthermore, this case study is the first report of a breast abscess caused by *Porphyromonas somerae*.

## Introduction

Compared with lactating women, mastitis and breast abscesses are rare in children and nonlactating women [1]. However, adolescent females and neonates before two months of age might develop mastitis and breast abscesses [1-3]. Mastitis is caused by a bacterial infection in the breast ductal system, which leads to breast abscesses if not promptly treated [1]. Common causes of breast infections include superficial breast injury, steroid use, immunodeficiencies, local skin infections, obesity, mammary duct ectasia, epidermoid cysts, and hidradenitis suppurativa [1]. In addition, in neonates, physiological hypertrophy of the newborn breast due to maternal estrogen and prolactin causes breast infections [2]. Here, we present a case of breast abscess in a three-year-old boy. The occurrence of pediatric breast abscesses in young boys after the neonatal period is extremely rare [3].

## Case presentation

A three-year-old boy was referred to our hospital for pain and redness in his right nipple. Nine days prior to his visit, he started experiencing pain in his right nipple. After three days, redness and pain persisted, prompting him to visit a clinic. A physical examination revealed a 4-5 cm diameter palpable mass in his right mammary gland without pus secretion. He was initially diagnosed with mastitis, and a first-generation cephalosporin was prescribed. However, the pain persisted. Two days before visiting our hospital, his right nipple became swollen. The day before visiting our hospital, the swelling worsened. Therefore, the clinic referred him to our hospital for further management. He had no relevant medical history. His birth history and neonatal metabolic screening were unremarkable, and his development had been normal. He did not have a cold or fever. In addition, he had no trauma recently and no operation history. His vaccinations were up to date, and he had no recent travel history. He was raised in an average Japanese household without any maltreatment and his family history was also unremarkable.

**Figure 1:**
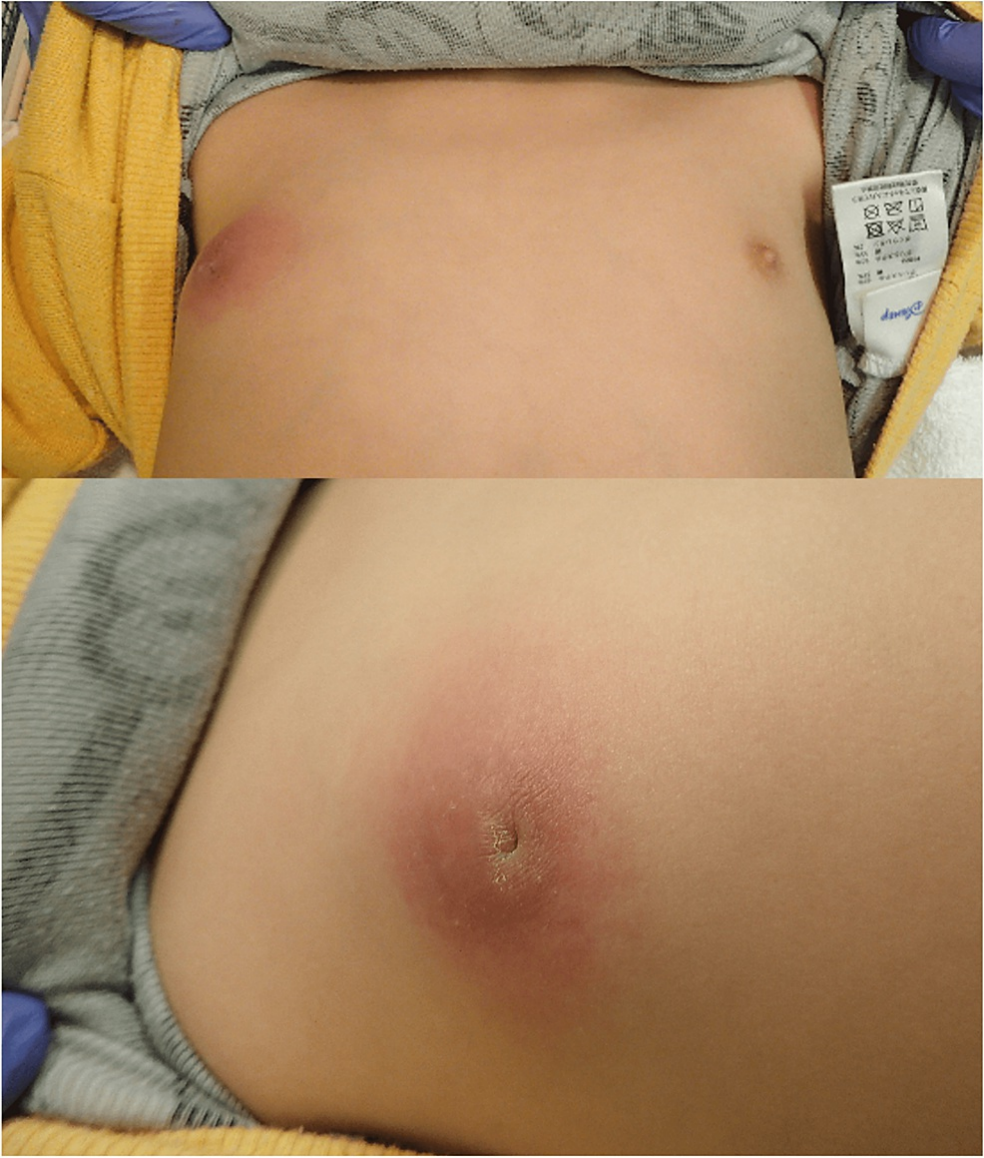
Redness of 4 cm in diameter on the right nipple. His right nipple was also inverted.

Upon arrival at the hospital, the patient exhibited stable vital signs and no fever. Physical examination revealed swelling, tenderness, and redness measuring 4 cm in diameter around his right nipple (Figure 1). However, his left nipple appeared normal without tenderness. No regional lymphadenopathies or signs of trauma were observed on his body. A complete blood count and basic metabolic panel yielded unremarkable results. Additional laboratory tests, including the C-reactive protein level, were within normal limits. Blood cultures also returned negative results.

**Figure 2:**
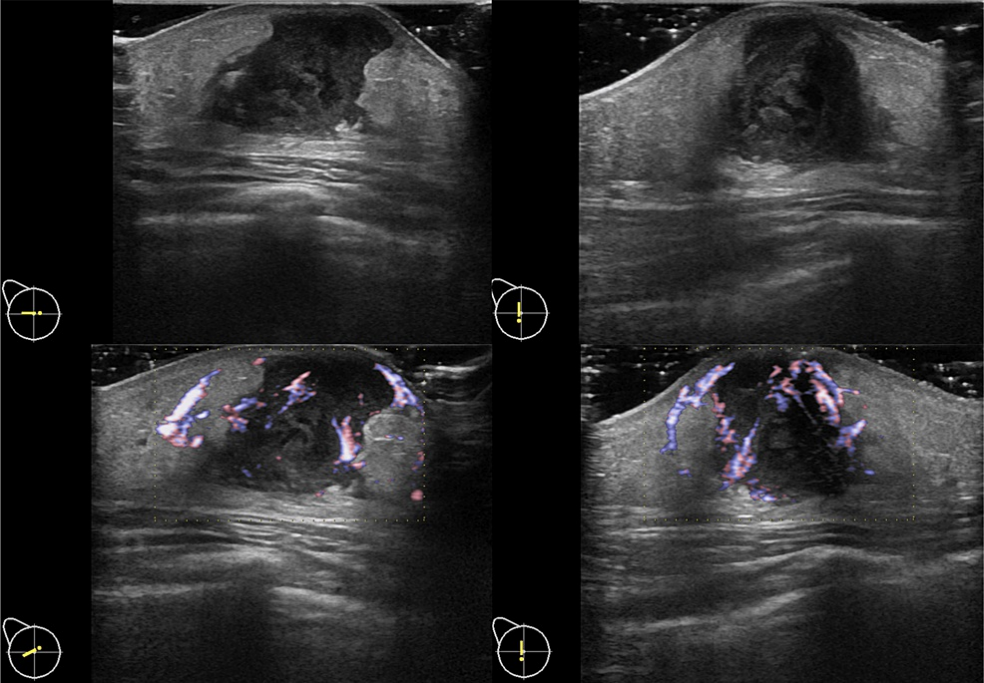
Ultrasonography showed a 26 mm × 18 mm irregularly shaped heterogeneous low echo-level area, poorly marginated, with a high blood flow signal suggesting a breast abscess.

The chest X-ray revealed no abnormalities; however, a chest ultrasound showed a right breast abscess measuring 26 mm × 18 mm (Figure 2). As a result, the patient was admitted to our hospital and started receiving intravenous cefazolin. On the third day of admission, incision and drainage (I&D) were performed, leading to a reduction in pain. After eight days of hospitalization, the patient was discharged and continued taking oral cefazolin for an additional two days. Subsequent follow-up visits at the clinic showed satisfactory progress. The results of the pus culture, obtained 21 days after the initial I&D, revealed the presence of three bacteria: *Peptoniphilus harei*, *Actinotignum sanguinis*, and *Porphyromonas somerae*. The detection of these organisms took a longer time due to the low bacterial load in the pus, likely influenced by the previous antibiotic treatment prescribed at the clinic. All organisms were identified using matrix-assisted laser desorption ionization-time of flight mass spectrometry. *P. harei* and *A. sanguinis* were found to be sensitive to penicillin and ceftriaxone, while all microorganisms were sensitive to cefmetazole. However, as symptoms reappeared one month after discharge, a repeat I&D was performed. Following the procedure, no recurrence was observed.

## Discussion

Breast infection is rare in boys beyond the neonatal age. In this particular case, the patient did not display any recurrent infections, indicating that he was immunocompetent. Additionally, he was not obese, and there was no evidence of breast or other site injuries. Therefore, the typical causes of breast infection for this age group did not apply to this case [1]. A case report has demonstrated that adolescent females with atopic dermatitis can experience breast abscesses [4]; however, the patient in this case did not have atopic dermatitis. A potential contributing factor could be his inverted right nipple. Since ductal infection often leads to breast abscesses, an inverted nipple could be susceptible to infection if not properly cleaned. The patient was born with a naturally inverted right nipple, while the left nipple was non-inverted. Maintaining good hygiene for inverted nipples may be important in young children to reduce the occurrence of breast abscesses. In the event of future recurrent infections, further examinations are recommended to rule out underlying conditions such as mammary duct ectasia, epidermoid cysts, and immunocompromised conditions like primary immunodeficiency, which can result in recurrent or unusual infections.

The most common bacteria in breast abscesses are *Staphylococcus aureus*, *Enterococcus*, *Streptococcus pyogenes*, *Pseudomonas*, and other gram-negative bacteria. Actinomycosis can cause mastitis in patients with nipple piercings [1]. Therefore, we started first-generation antibiotics for his treatment. Our case showed *P. harei*, *A. sanguinis*, and *P. somerae* in the aspirated pus culture, which was rarely observed previously [5,6]. Besides, this is the first report of a breast abscess caused by *P. somerae*, a gram-negative and anaerobic bacteria of the genus *Porphyromonas*. One study reported that this pathogen was isolated from a human leg ulcer in the United States [7]. In this case, the possible reasons for detecting the rare bacteria are that the breast abscess infection in young boys is rare, and the infection process is not usually due to stocked dirt, including the bacteria in inverted nipples.

In the present case, we suspected that the cause of the infection was cefazolin-sensitive organisms since it was an abscess. However, retrospectively, our patient recovered from the breast abscess by performing I&D rather than only administering antibiotics. The quality of patient care requires careful consideration before performing I&D because of the cosmetic and functional implications for the breast [8]. Therefore, noninvasive treatments, including antibiotics, are the first-line treatment. In addition, physicians should consider the anesthetic risk for surgical procedures in children. However, we considered the possibility of treatment failure and performed additional procedures without hesitation.

## Conclusions

Although breast abscesses are rare in adolescents and neonates, we still need to consider the possibility of an underlying condition. Our case highlighted that an inverted nipple could cause a breast infection, and hygiene of inverted nipples might be important. Additionally, if the prognosis of antibiotic treatment is unsatisfactory, we should consider performing I&D because of the possibility of unusual bacteria causing infection, such as *Peptoniphilus harei*, *Actinotignum sanguinis*, and *Porphyromonas somerae*.

## References

[1] Mareti E, Vatopoulou A, Spyropoulou GA (2021). Breast disorders in adolescence: a review of the literature. Breast Care (Basel).

[2] Jean Bertrand KA, Rose NK, Franck LG, Célestin BA, Ibrahim T, N'gouan Constance BU (2022). Mastitis and breast abscess in newborns and infants. Afr J Paediatr Surg.

[3] Ansari E, Harper MB, Landscahft A, Kimia R, Lynn A, Ozonoff A, Kimia AA (2021). Bacteriology of pediatric breast abscesses beyond the neonatal period. Am J Emerg Med.

[4] Park SM, Choi WS, Yoon Y (2018). Breast abscess caused by Staphylococcus aureus in 2 adolescent girls with atopic dermatitis. Korean J Pediatr.

[5] Le Bihan A, Ahmed F, O'Driscoll J (2019). An uncommon cause for a breast abscess: Actinomyces turicensis with Peptoniphilus harei. BMJ Case Rep.

[6] Calatrava E, Borrego J, Cobo F (2019). Breast abscess due to Trueperella bernardiae and Actinotignum sanguinis. Rev Esp Quimioter.

[7] Summanen PH, Durmaz B, Väisänen ML (2005). Porphyromonas somerae sp. Nov., a pathogen isolated from humans and distinct from Porphyromonas levii. J Clin Microbiol.

[8] Conde DM (2015). Treatment approach for breast abscess in nonlactating adolescents. Int J Gynaecol Obstet.

